# BML-111 Reduces Neuroinflammation and Cognitive Impairment in Mice With Sepsis via the SIRT1/NF-κB Signaling Pathway

**DOI:** 10.3389/fncel.2018.00267

**Published:** 2018-08-21

**Authors:** Shangwen Pan, Yan Wu, Lei Pei, Shengnan Li, Limin Song, Haifa Xia, Yaxin Wang, Yuan Yu, Xiaobo Yang, Huaqing Shu, Jiancheng Zhang, Shiying Yuan, You Shang

**Affiliations:** ^1^Department of Critical Care Medicine, Union Hospital, Tongji Medical College, Huazhong University of Science and Technology, Wuhan, China; ^2^Department of Neurology, Union Hospital, Tongji Medical College, Huazhong University of Science and Technology, Wuhan, China; ^3^Department of Neurobiology, Tongji Medical College, Huazhong University of Science and Technology, Wuhan, China; ^4^Department of Anesthesiology, Union Hospital, Tongji Medical College, Huazhong University of Science and Technology, Wuhan, China

**Keywords:** sepsis, neuroinflammation, cognitive impairment, BML-111, SIRT1, NF-κB

## Abstract

Sepsis is a life-threatening state of organ dysfunction caused by infection and which can induce severe neurological disorders that lead to neuroinflammation and cognitive impairment. Inflammation has been reported to cause neuronal apoptosis in sepsis, which can finally lead to cognitive impairment. Previous studies have suggested that BML-111 can exhibit anti-inflammatory and proresolution activities. Additionally, silent information regulator 1 (SIRT1) can inhibit the NF-κB signaling pathway in an inflammation state. However, the role of the SIRT1/NF-κB signaling pathway in the protective effects of BML-111 against sepsis-induced neuroinflammation and cognitive impairment remains unclear. This study aimed to determine the effects of BML-111 on neuroinflammation and cognitive impairment induced by sepsis. Male C57BL/6J mice were subjected to cecal ligation and puncture (CLP) or a sham operation. BML-111 was administered via intracerebroventricular injection (0.1 mg/kg) immediately after CLP. Boc-2 (50 μg/kg) was administered intracerebroventricularly 30 min before CLP, and EX527 (10 μg) was administered every 2 days for a total of three times before CLP, also intracerebroventricularly. Some of the surviving mice underwent open-field, novel-object-recognition, and fear-conditioning behavioral tests at 7 days after surgery. Some of the other surviving mice were killed at 24 h after surgery to assess synaptic damage (PSD95 and Synapsin1), markers of inflammation [tumor necrosis factor alpha (TNF-α) and interleukin (IL)-1β], cytoplasmic p65, nuclear p65, Ac- NF-κB and SIRT1. At 48 h after CLP, TUNEL and glia-activation by immunofluorescence investigations were performed on a separate cohort of surviving animals. The results suggested that sepsis resulted in cognitive impairment, which was accompanied by the decreased the expression of PSD95 and Synapsin1, increased amount of TUNEL-positive cells and the activation of glias, increased production of TNF-α and IL-1β, increased expression of nuclear p65, Ac- NF-κB, and decreased expression of SIRT1 and cytoplasmic p65. It is especially notable that these abnormalities could be reduced by BML-111 treatment. EX527, an SIRT1 inhibitor, abolished the effects of BML-111. These results demonstrate that BML-111 can reduce the neuroinflammation and cognitive impairment induced by sepsis via SIRT/NF-κB signaling pathway.

## Introduction

Sepsis is defined as a life-threatening state of organ dysfunction caused by a dysregulated host response to infection, and it is the main reason for mortality in hospitalized patients (Singer et al., 2016). Sepsis is a systemic response, and so many organs such as the heart, kidney, liver, and brain are involved (Rudiger and Singer, 2007; Adam et al., 2013; Chen et al., 2014; Liu A. et al., 2015). The brain is affected earlier and more frequently than the other organs (Dal-Pizzol et al., 2014), and this leads to neuroinflammation and cognitive impairment (Wu et al., 2015). During sepsis, periphery cytokines disrupt the blood brain barrier and activate microglias, which then markedly increase the production of proinflammatory cytokines, chemokines, and reactive oxygen species in the brain. These increased levels of cytokines exacerbate the inflammation reaction and induce neuronal apoptosis, which can finally lead to cognitive impairment (Wu et al., 2015).

Uncontrolled inflammation plays a crucial role in neuronal apoptosis and subsequent cognitive impairment in sepsis, which has prompted extensive exploration the classical regulator of inflammation, NF-κB. After being activated in sepsis, NF-κB increases the expression of proinflammatory cytokines such as nitric oxide, tumor necrosis factor alpha (TNF-α), and interleukin (IL)-1β, and amplifies the inflammatory response (Li et al., 2016; Qin et al., 2016). Furthermore, previous studies have found that the inflammatory response in the brain during sepsis can induce neuronal apoptosis and subsequent cognitive impairment (Wu et al., 2015). Although inhibiting inflammation could improve the outcome of cell death and cognitive impairment in sepsis (Wu et al., 2015; Sui et al., 2016), the relationships between NF-κB, sepsis-induced neuroinflammation and cognitive impairment is still need to be elucidated.

Silent information regulator 1 (SIRT1), is a conserved deacetylase that is dependent on nicotinamide adenine dinucleotide and reportedly involved in sepsis (Zhao et al., 2015; Opal et al., 2016). SIRT1 exerts protective properties during the development of sepsis, and some of the underlying mechanisms have already been identified (Zhao et al., 2015; McCreath et al., 2016; Opal et al., 2016). Although SIRT1 is critical for the neuronal degeneration and cognitive decline that occurs in aging disorders (Ng et al., 2015) and protects the central nervous system via the negative regulation of NF-κB (Hernandez-Jimenez et al., 2013; Kauppinen et al., 2013), the protective effects of SIRT1 on sepsis-induced neuroinflammation and subsequently cognitive impairment remains unclear.

Lipoxin A4 (LXA4), an endogenous molecule that exhibits anti-inflammatory and proresolution properties, is biosynthesized from arachidonic acid by lipoxygenase. After combining with the formyl peptide receptor 2/lipoxin A4 receptor (FPR2/ALX) of neutrophils, monocytes, astrocytes, and microglias (Chiang et al., 2006; Svensson et al., 2007; Wang et al., 2011), LXA4 limits the recruitment of neutrophils, increases the production of anti-inflammatory mediators, and promotes the clearance of inflammatory debris. However, native LXA4 is rapidly biosynthesized and inactivated, and so it is necessary to synthesize stable and powerful analogs (Chiang et al., 2000). BML-111, 5(S), 6(R), 7-trihydroxyheptanoic acid methyl ester, is a synthetic ALX agonist, which reportedly inhibits neutrophil recruitment and peripheral inflammation (Lee et al., 1991; Gong J. et al., 2012; Li et al., 2013; Kong et al., 2015). It has been demonstrated that BML-111 exerts anti-inflammatory effects in the cerebral cortex and maintains the integrity of the blood–brain barrier after ischemic stroke (Hawkins et al., 2014). BML-111 has also been reported to protect the intestinal mucosal barrier and lungs during sepsis (Liu H. et al., 2015; Tang et al., 2016).

However, the protective effects of BML-111 in sepsis-induced neuroinflammation and cognitive impairment are unknown, and so in this study we hypothesized that BML-111 reduces neuroinflammation and cognitive impairment induced by sepsis via the SIRT1/NF-κB signaling pathway.

## Materials and Methods

### Ethics Statement

All animal experiments in this study were performed in accordance with the Guide for the Care and Use of Laboratory Animals of Tongji Medical College. The *in vivo* protocols were approved by the Committee of Experimental Animals of Tongji Medical College of Huazhong University of Science and Technology. Every effort was made to minimize animal suffering.

### Animals and the Cecal Ligation and Puncture Model

All of the male C57BL/6J mice used in this study were purchased from Beijing HFK BIOSCIENCE and weighed between 22 and 25 g (6–8 weeks old). All mice were kept under pathogen-free conditions at a temperature of around 22°C and a 12-h of light/dark cycle with free access to food and water. The mice were fasted for at least 8 h, but water was allowed *ad libitum* before performing cecal ligation and puncture (CLP).

Animals were subjected to CLP as reported previously with some modifications (Toscano et al., 2011; Zhao et al., 2015). In brief, under pathogen-free conditions, mice were anesthetized with an intraperitoneal injection of 2% sodium pentobarbital at 80 mg/kg, and then a 1-cm abdominal midline incision was made to carefully expose the cecum and related intestines. The cecum was ligated below the ileocecal valve without causing obstruction and a 20-gauge needle was used to puncture the cecum twice. A small amount of stool was gently squeezed through the puncture sites into the peritoneal cavity. The bowel was then repositioned in the abdomen as carefully as possible, and the peritoneum and skin were sutured using a sterile 4.0 silk sutures. The mice in the sham-operated group underwent the same operation but without CLP being performed. Animals in all of the groups received basic normal saline resuscitation (50 ml/kg) injected subcutaneously immediately after surgery, and then the rectal temperature was kept at 36.5–37.5°C with the aid of a homoeothermic blanket. All steps of the surgical procedure were finished within 10 min and the animals were put back to their cages with free access to food and water after being revived.

BML-111 (Enzo Life Sciences, NY, United States), Boc-2 (Genscript, Piscataway, NJ, United States), and EX527 (an inhibitor of SIRT1; Sigma-Aldrich, St. Louis, MO, United States) were first dissolved in dimethyl sulfoxide (DMSO) (Sigma) and then diluted in normal saline (the final DMSO concentration was 1%). Prior to drug administration, the lateral ventricle injection site (0.3 mm posterior, 1.0 mm lateral, and 2.5 mm ventral to the bregma) was confirmed by injecting of Evans blue dye with the help of a stereotaxis instrument (RWD, Shenzhen, China). The dose response relationship was determined by injecting 2 μl of BML-111 at 0.01 and 0.1 mg/kg intracerebroventricularly via a microsyringe with a 28-gauge stainless-steel needle at a rate of 0.5 μl/min (Li et al., 2010) immediately after the surgery to determine the optimal dose for reducing the cognitive impairment induced by sepsis. For the vehicle groups, the same volume (2 μl) of 1% DMSO was given immediately after the surgery. Boc-2 and EX527 were used after the dose response relationship had been investigated. Boc-2 at a dose of 50 μg/kg (2 μl) (Gong J. et al., 2012) or the same volume (2 μl) of 1% DMSO was injected intracerebroventricularly at 30 min prior to the CLP operation. 10 μg EX527 (2 μl) (Yang et al., 2015) or the same volume (2 μl) of vehicle (1% of DMSO) was administered every 2 days for a total of three times prior to performing the CLP operation.

### Behavioral and Cognitive Tests

The survivors underwent three behavioral tasks at 7 days after the surgery: open-field test, novel-object-recognition test, and fear-conditioning.

### Open-Field Test

The open field test was used to record the exploratory behaviors of the mice. At 7 days after CLP, the mice were placed at the center of a white plastic chamber (50 cm × 50 cm × 40 cm; Tai Meng Technology, Chengdu, China). Their move distance and time were recorded over a 5-min period. After each test, all inner faces of the device were cleaned up with 75% alcohol to avoid interanimal effects.

### Novel-Object-Recognition Test

The novel-object-recognition test was executed as reported previously with some modifications (Leger et al., 2013; Wei et al., 2015). In brief, for training, animals were placed in an open-field and allowed to explore two novel objects (A and B) for 5 min. The test was carried out 24 h after this training period, during which the same mouse was allowed to explore the field for 5 min with familiar object A and a novel object C. The time spent in sniffing, touching, and orienting to each object was recorded as the exploration time. The recognition index was defined as TB/(TA + TB) or TC/(TA + TC), where TA, TB, and TC are the times spent exploring the objects A, B, and C, respectively.

### Fear-Conditioning Test

The fear-conditioning test was employed as reported previously for assessing associative learning and memory (Jin et al., 2016). Briefly, the mice were placed into a chamber (33 cm × 33 cm × 35 cm) and allowed to adapt to the novel environment for 5 min before training. During the training day, the mice received 5-min trials that started with a 30-s tone, followed by a 2-s foot shock (1 mA) and then a 30-s recovery interval, which was repeated three times. The contextual and tone-conditioning tasks were performed 24 h later. To test contextual conditioning fear, mice were returned to the same chamber without a foot shock or tone. To test tone-dependent conditioning fear, some changes were made to the visual, tactile, and olfactory cues in order to present the mice with a new chamber environment. The mice were then placed in the chamber for 5 min without foot shock.

### Western Blotting

Twenty-four hours after the CLP, a cohort of the surviving animals was anesthetized with sodium pentobarbital. After the animals were decapitated, the cortex and hippocampus were isolated. Some of the tissues were homogenized with radioimmune precipitation assay buffer for detecting total proteins, and others were homogenized with the NE-PER Nuclear and Cytoplasmic Extraction Reagent kit (Pierce Biotechnology, Rockford, IL, United States) for detecting cytoplasmic and nuclear proteins, after which a protease inhibitor cocktail and phosphatase inhibitors (Roche Molecular Biochemicals, Inc., Mannheim, Germany) were added. To detect the variation of postsynaptic density protein 95 (PSD95) and Synapsin1, cortex and hippocampus tissue samples were homogenized in 200 μl of 0.32 M sucrose buffer (mM) (10 sucrose, 10 HEPES, pH 7.4) containing a protease inhibitor cocktail. Samples were centrifuged (1000 × *g* for 10 min at 4°C) to yield the nuclear enriched pellet and the S1 fraction. The nuclear enriched pellet was discarded and the S1 fraction was centrifuged (12,000 × *g* for 20 min at 4°C) to obtain the supernatant (S2; microsomes and cytosol) and pellet (P2; crude synaptosomal membranes) fractions. The S2 fraction was discarded, and the P2 synaptosomal pellet was resuspended in 100 μl of 4 mM HEPES buffer (mM) (4 HEPES, 1 EDTA, pH 7.4) and then centrifuged again (12,000 × *g* for 20 min at 4°C). The resuspension and centrifugation procedures were repeated, and the resulting pellet was resuspended with buffer A (mM) (20 HEPES, 100 NaCl, 0.5% Triton, pH 7.2) and then rotated slowly (15 min, 4°C), followed by centrifugation (12,000 × *g* for 20 min at 4°C). The supernatant (Triton-soluble NP fraction) containing non-PSD95 membranes was discarded. The pellet was resuspended in 120 μl of buffer B (20 mM HEPES, 0.15mM NaCl, 1% Triton-X, 1% deoxycholic acid, 1% SDS, and 1 mM DTT, pH 7.5), followed by gentle rotation (1 h at 4°C) and centrifugation (10,000 × *g* for 15 min at 4°C). The pellet was discarded and the supernatant (the Triton-X insoluble PSD95 fraction) was retained. The obtained PSD95 samples were stored at −80°C until use (Pei et al., 2015). Proteins were separated by 10% SDS/polyacrylamide gel electrophoresis before being transferred to PVDF membranes. The membranes were blocked with 5% non-fat milk in TBST, and immunohistochemistry was performed with primary antibodies against PSD95, Synapsin1, NF-κB p65 (p65), SIRT1 (1:1000; from Abcam, Cambridge, MA, United States), acetylated-NF-κB p65 (Ac- NF-κB) (1:1000; from Cell Signaling Technology, Boston, MA, United States), β-actin, and Lamin B1 (1:1000; Santa Cruz, CA, United States) at 4°C overnight. The membranes were then incubated with the respective secondary antibodies for 1 h. The proteins were detected using enhanced chemiluminescence (Thermo Fisher Scientific, Waltham, MA, United States), with β-actin was used as a loading control. The intensity of the protein bands was ultimately analyzed using the Image J software (version 1.45s, NIH, Bethesda, MD, United States).

### Tunel Assay

After 48 h after CLP, the deoxynucleotidyl-transferase-mediated dUTP nick-end-labeling (TUNEL) assay was used to detect cell death in accordance with the manufacturer’s instructions (Roche Molecular Biochemicals, Inc., Mannheim, Germany). Slices were deparaffinized and then incubated in the TUNEL reaction mixture for 1 h at 37°C. Images were captured with an Olympus IX71 fluorescence microscope (Olympus, Tokyo, Japan) by an investigator who was blinded to the identities of the samples.

### Immunofluorescence Assay

At the point of 48 h after the surgery, the surviving animals were anesthetized and then perfused via the left cardiac ventricle with phosphate-buffered saline (PBS), followed by 4% paraformaldehyde. The brains were then carefully removed and postfixed in 4% paraformaldehyde for an additional 72 h. The tissues were embedded in paraffin according to standard protocols. Paraffin-embedded 5-μm-thick brain tissue sections were permeabilized with PBS containing 1% Triton X-100 for 10 min, blocked with 10% normal goat serum for 1 h, and then incubated overnight at 4°C with the following primary antibodies: rabbit-anti-Iba1 (1:200; Wako, Japan) and mouse-anti-GFAP (1:300; Cell Signaling Technology, Boston, MA, United States). After washing three times with PBS, the sections were incubated with IFKine Green AffiniPure Donkey Anti-Rabbit IgG and Donkey Anti-Mouse IgG (1:200; Abbkine, CA, United States) for 1 h at room temperature. Images were captured with the aid of a laser-scanning confocal microscope (LSM 510, Carl Zeiss, Germany) by an investigator who was blinded to the identities of the samples.

### Enzyme-Linked Immunosorbent Assay

For the enzyme-linked immunosorbent assay (ELISA), tissue samples were collected from the cortex and hippocampus of surviving animals at 24 h after the surgery. The levels of TNF-α and IL-1β were quantified using ELISA kits (Boster Biological Technology, Wuhan, China) according to the manufacturer’s instructions. Readings from each sample were normalized to the protein concentration.

### Statistical Analyses

GraphPad Prism software (version 5 for Windows, San Diego, CA, United States) was used for all statistical analyses. All values were expressed as mean ± SEM. The move time and distance, fear-conditioning data, Western-blot results, TUNEL positive cells, microglia cells, astrocytes cells and ELISA data were analyzed using one-way ANOVA with the Student–Newman–Keuls tests. The novel-object-recognition data were analyzed using two-way ANOVA with the Bonferroni tests. Statistical significance was accepted at *P* < 0.05.

## Results

### Sepsis-Induced Cognitive Dysfunction Was Reduced by BML-111 at the Optimal Dose

**FIGURE 1:**
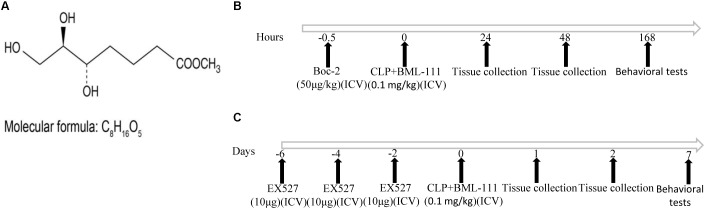
Molecular formula and structure of BML-111 **(A)** and a schematic representation of the experiments **(B,C)**.

**FIGURE 2:**
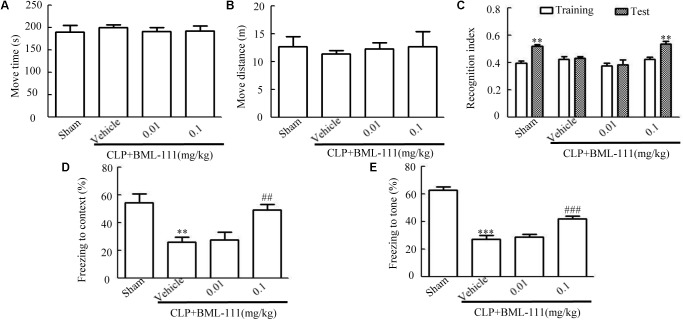
Influences of BML-111 at different doses on cognitive dysfunction in mice with sepsis. BML-111 was injected intracerebroventricularly at doses of 0.01 and 0.1 mg/kg immediately after performing CLP. **(A,B)** No significant difference was observed in the move time and distance parameters in the open-field test. **(C–E)** Effects of BML-111 at different doses on novel-object-recognition and fear-conditioning tests results. Data were shown as the means ± SEM (*n* = 10 surviving mice per group). ^∗∗^*P* < 0.01 represents Test vs. Training in **(C)**. ^∗∗^*P* < 0.01, ^∗∗∗^*P* < 0.001 represent CLP + Vehicle vs. Sham and ^##^*P* < 0.01, ^###^*P* < 0.001 represents CLP + 0.1 mg/kg BML-111 vs. CLP + Vehicle in **(D,E)**.

The structure of BML-111 (Cyman, MI, USA) and its molecular formula (Enzo, NY, United States) were presented in **Figure 1A**. The time chart of the experiment was shown in **Figures 1B,C**. As indicated in **Figures 2C–E**, the investigation of the dose response relationship showed that BML-111 at a dose of 0.1 mg/kg was effective at increasing the recognition index and freezing behaviors when mice were performing the novel-object-recognition and fear-conditioning tests. A BML-111 dose of 0.1 mg/kg was therefore used in the subsequent experiments. In addition, the move time and distance did not differ in any of the groups (**Figures 2A,B**), which demonstrates that BML-111 treatment and CLP did not influence the motor or exploratory activity.

### Sepsis-Induced Cognitive Dysfunctions Was Reduced by BML-111 Treatment

**FIGURE 3:**
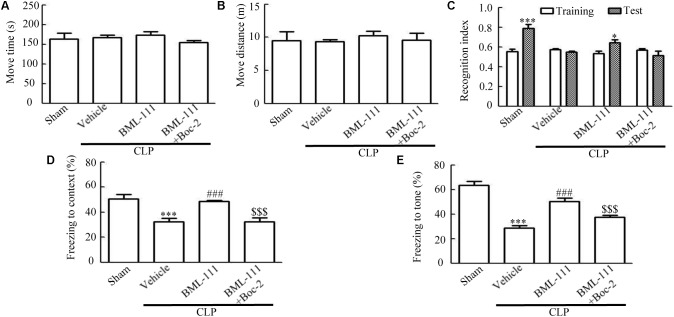
BML-111 treatment reduces cognitive dysfunction in mice with sepsis. **(A,B)** The move time and distance in the open-field test did not differ among the four groups. **(C)** Sepsis did not result in the recognition index for the new object differing from that for the familiar one, whereas that for the new object in the sham group differed significantly from that for the familiar one. BML-111 treatment reversed the decrease in the recognition index in the CLP mice, and Boc-2 inhibited the effects of BML-111. **(D,E)** The freezing times in the contextual and tone-conditioning tasks were significantly decreased in the CLP + vehicle group compared with the sham group. BML-111 treatment reversed this decrease, and Boc-2 suppressed the effects of BML-111. Data were shown as the means ± SEM (*n* = 10 surviving mice per group). ^∗^*P* < 0.05, ^∗∗∗^*P* < 0.001 represent Test vs. Training in **C**. ^∗∗∗^*P* < 0.001 represents CLP + Vehicle vs. Sham, ^###^*P* < 0.001 represents CLP + BML-111 vs. CLP + Vehicle, ^$$$^*P* < 0.001 represents CLP + BML-111 + Boc-2 vs. CLP + BML-111 in **(D,E)**.

**Figures 3A,B** indicate that there were no differences in the move time and distance among the four groups in the open-field test (*P* > 0.05), which is consistent with our previous results. In the novel-object-recognition test (**Figure 3C**), mice in the sham group spent more time investigating the new object, leading to the recognition index differing significantly between the new object and the familiar one (*P* < 0.001). Mice in the CLP + vehicle group spent less time investigating the new object, resulting in the recognition index not differing between the new object and the familiar one (*P* > 0.05), which suggests that sepsis resulted in cognitive impairments. Administering BML-111 reduced the cognitive impairment, which presented as an improvement in recognition index compared with that for the familiar object (*P* < 0.05). In addition, pretreatment with Boc-2 (which is an antagonist of ALX) before CLP and BML-111 treatment can abolish the effects of BML-111 (*P* > 0.05, **Figure 3C**). When the mice performed the fear-conditioning test (**Figures 3D,E**), the freezing times in the contextual and tone-conditional tasks were significantly lower in the CLP + vehicle group than in the sham group (*P* < 0.001, **Figure 3D**; and *P* < 0.001, **Figure 3E**). It is important to emphasize that administering BML-111 immediately after performing CLP significantly increased the freezing behaviors when compared with CLP + vehicle group (*P* < 0.001, **Figure 3D**; and *P* < 0.001, **Figure 3E**). Administering Boc-2 before CLP and BML-111 treatment significantly decreased the freezing time compared to that in the BML-111-treated group (*P* < 0.001, **Figure 3D**; and *P* < 0.001, **Figure 3E**).

### Sepsis-Induced Synaptic and Neuronal Damage Were Ameliorated by BML-111 Administration

**FIGURE 4:**
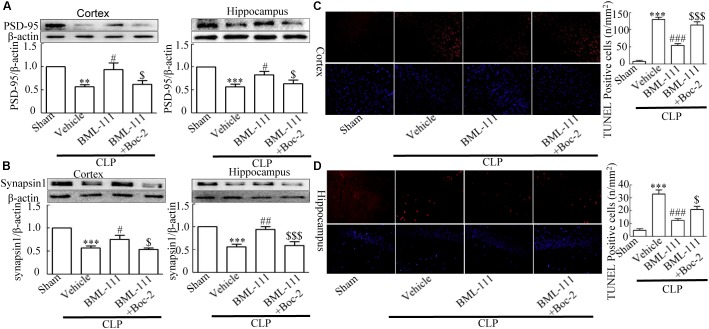
BML-111 treatment reduces the decreases in PSD95, Synapsin1 and TUNEL-positive cells. PSD95 and Synapsin1 **(A,B)** in the cortex and hippocampus were significantly reduced in CLP mice compared with the sham group. BML-111 treatment increased the expression of PSD95 and Synapsin1 in CLP mice, and Boc-2 decreased the effects of BML-111. **(C,D)** The number of TUNEL-positive cells was significantly increased in CLP mice compared with the sham group. BML-111 treatment reversed this variation in CLP mice, and Boc-2 reduced these effects. Scale bars indicate 20 mm. Data were shown as the means ± SEM (*n* = 6 surviving mice in **A** cortex, *n* = 5 surviving mice in **A** hippocampus, *n* = 6 surviving mice in **B** cortex, *n* = 4 surviving mice in **B** hippocampus, *n* = 3 surviving mice per group in **C,D**). ^∗∗^*P* < 0.01, ^∗∗∗^*P* < 0.001 represent CLP + Vehicle vs. Sham, ^#^*P* < 0.05, ^##^*P* < 0.01, ^###^*P* < 0.001 represents CLP + BML-111 vs. CLP + Vehicle, ^$^*P* < 0.05, ^$$$^*P* < 0.001 represents CLP + BML-111 + Boc-2 vs. CLP + BML-111 in this Figure.

Western blotting was applied to the cortex and hippocampus tissue samples to detect synaptic-associated PSD95 and Synapsin1. CLP significantly reduced the expression levels of PSD95 and Synapsin1 compared to the sham group (*P* < 0.01, **Figure 4A**, cortex; *P* < 0.001, **Figure 4A**, hippocampus; *P* < 0.001, **Figure 4B**, cortex; and *P* < 0.001, **Figure 4B**, hippocampus). When BML-111 was administered to the CLP mice, the protein levels of PSD95 and Synapsin1 were significantly increased in the cortex and hippocampus (*P* < 0.05, **Figure 4A**, cortex; *P* < 0.05, **Figure 4A**, hippocampus; *P* < 0.05, **Figure 4B**, cortex; and *P* < 0.01, **Figure 4B**, hippocampus). After administering Boc-2 (an antagonist of ALX), the protein levels of PSD95 and Synapsin1 were significantly decreased in the cortex and hippocampus (*P* < 0.05, **Figure 4A**, cortex; *P* < 0.05, **Figure 4A**, hippocampus; *P* < 0.05, **Figure 4B**, cortex; and *P* < 0.001, **Figure 4B**, hippocampus). Apoptosis of cortex and hippocampus cells was subsequently detected using the TUNEL assay. Compared with the sham group, large numbers of TUNEL-positive cells were observed in the cortex and hippocampus of mice subjected to CLP (*P* < 0.001, **Figure 4C**; *P* < 0.001, **Figure 4D**). BML-111 decreased the number of TUNEL-positive cells (*P* < 0.001, **Figure 4C**; *P* < 0.001, **Figure 4D**), while pretreatment with Boc-2 increased the number of TUNEL-positive cells in the cortex and hippocampus compared with BML-111 treatment alone (*P* < 0.001, **Figure 4C**; *P* < 0.05, **Figure 4D**).

### Sepsis Induced Glia Activation Was Inhibited by BML-111 Administration

**FIGURE 5:**
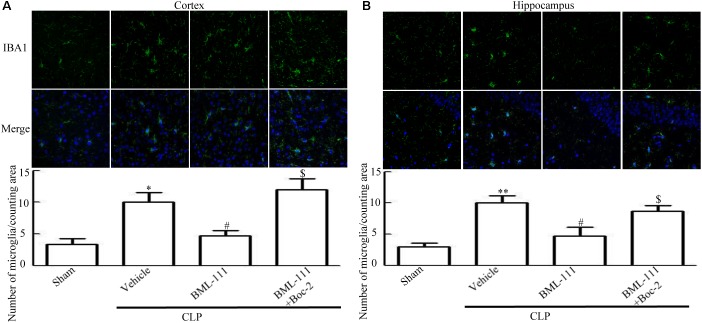
BML-111 treatment reduces the activation of microglias in CLP mice. **(A,B)** Activated-microglia cells were significantly increased in the CLP + vehicle group compared with the sham group. BML-111 treatment down-regulated the activated cells compared with the CLP + vehicle group, and Boc-2 reduced the effects of BML-111. Scale bars indicate 50 μm (*n* = 3 surviving mice per group per experiment). Data were shown as the means ± SEM. ^∗^*P* < 0.05, ^∗∗^*P* < 0.01 represent CLP + Vehicle vs. Sham, ^#^*P* < 0.05 represents CLP + BML-111 vs. CLP + Vehicle, ^$^*P* < 0.05 represents CLP + BML-111 + Boc-2 vs. CLP + BML-111.

**FIGURE 6:**
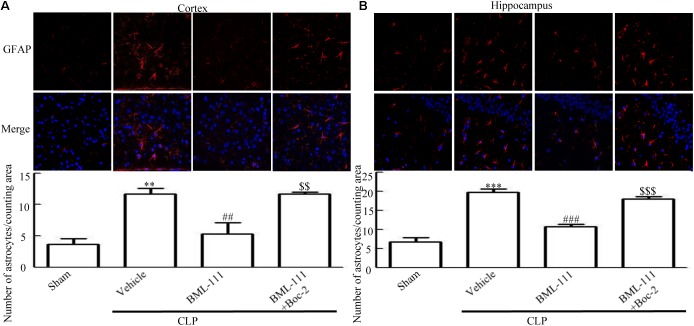
BML-111 treatment reduces the activation of astrocyte in mice with sepsis. **(A,B)** CLP up-regulated the GFAP-positive cells significantly compared with the sham group. BML-111 treatment decreased the number of positive cells compared with the CLP + vehicle group, and Boc-2 reduced the effects of BML-111. Scale bars indicate 50 μm (*n* = 3 surviving mice per group per experiment). Data were shown as the means ± SEM. ^∗∗^*P* < 0.01, ^∗∗∗^*P* < 0.001 represent CLP + Vehicle vs. Sham, ^##^*P* < 0.01, ^###^*P* < 0.001 represent CLP + BML-111 vs. CLP + Vehicle, ^$$^*P* < 0.01, ^$$$^*P* < 0.001 represent CLP + BML-111 + Boc-2 vs. CLP + BML-111.

As reported previously, microglias and astrocytes are activated during sepsis (Semmler et al., 2005; Michels et al., 2014). To examine the effects of BML-111 on the activation of glias, Iba1 and GFAP were used as markers of microglias and astrocytes. As **Figures 5**,**6** shown, large numbers of Iba1- and GFAP-positive cells were observed in the cortex and hippocampus of CLP mice, whereas very few such cells were visible in the sham-operated group (*P* < 0.05, **Figure 5A**; *P* < 0.01, **Figure 5B**; *P* < 0.01, **Figure 6A**; *P* < 0.001, **Figure 6B**). It should be emphasized that BML-111 decreased the numbers of Iba1- and GFAP-positive cells in the cortex and hippocampus relative to the CLP + vehicle group (*P* < 0.05, **Figure 5A**; *P* < 0.05, **Figure 5B**; *P* < 0.01, **Figure 6A**; *P* < 0.001, **Figure 6B**). Administering Boc-2 before CLP and BML-111 treatment increased the numbers of Iba1- and GFAP-positive cells in the cortex and hippocampus relative to the BML-111- treatment group. (*P* < 0.05, **Figure 5A**; *P* < 0.05, **Figure 5B**; *P* < 0.01, **Figure 6A**; *P* < 0.001, **Figure 6B**).

### Sepsis-Induced Neuroinflammation and Activation of NF-κB Were Reduced by BML-111 Injections

**FIGURE 7:**
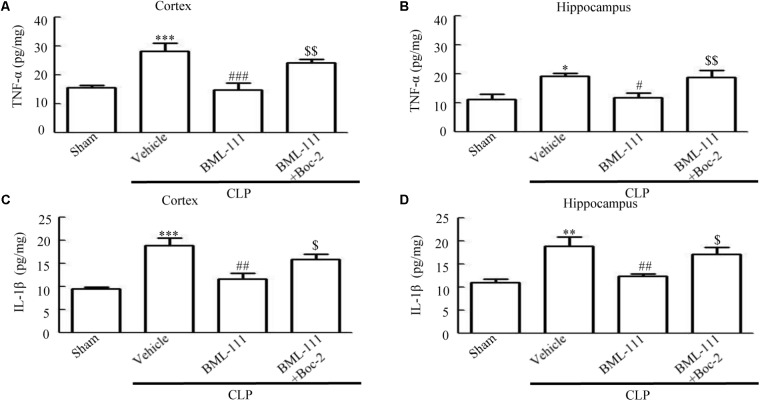
BML-111 treatment reduces the expression of inflammatory cytokines. **(A–D)** The TNF-α and IL-1β levels were significantly increased in CLP mice compared with the sham group. BML-111 treatment reduced the cytokine levels when compared with the CLP + vehicle group, and Boc-2 reduced the effects of BML-111. Data were shown as the means ± SEM (*n* = 8 surviving mice in **A,B**, *n* = 6 surviving mice in **C,D**). ^∗^*P* < 0.05, ^∗∗^*P* < 0.01, ^∗∗∗^*P* < 0.001 represent CLP + Vehicle vs. Sham, ^#^*P* < 0.01, ^##^*P* < 0.01, ^###^*P* < 0.001 represent CLP + BML-111 vs. CLP + Vehicle, ^$^*P* < 0.05, ^$$^*P* < 0.01 represent CLP + BML-111 + Boc-2 vs. CLP + BML-111.

TNF-α and IL-1β were measured to assess the presence of neuroinflammation in the cortex and hippocampus of the sepsis survivors. As the results in **Figure 7** show, the presence of sepsis up-regulated the proinflammatory mediators, TNF-α and IL-1β, in the cortex and hippocampus relative to the sham group (*P* < 0.001, **Figure 7A**; *P* < 0.05, **Figure 7B**; *P* < 0.001, **Figure 7C**; and *P* < 0.01, **Figure 7D**). Administering BML-111 after CLP significantly reduced the increases in these mediators relative to mice subjected to CLP (*P* < 0.001, **Figure 7A**; *P* < 0.05, **Figure 7B**; *P* < 0.01, **Figure 7C**; and *P* < 0.01, **Figure 7D**). Administering Boc-2 before CLP and BML-111 treatment was effective at increasing the expression levels of TNF-α and IL-1β (*P* < 0.01, **Figure 7A**; *P* < 0.01, **Figure 7B**; *P* < 0.05, **Figure 7C**; and *P* < 0.05, **Figure 7D**). Meanwhile, the level of TNF-α in the blood was unchanged when BML-111 or Boc-2 was administered before CLP and BML-111 treatment (*P* > 0.05, **Supplementary Figure S1**).

**FIGURE 8:**
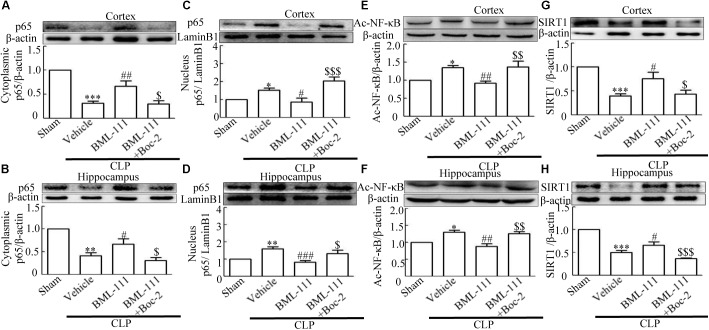
BML-111 treatment significantly inhibits NF-κB and increases the expression of SIRT1. **(A,B)** Sepsis significantly reduced the expression levels of cytoplasmic p65 in the cortex and hippocampus relative to the sham group. BML-111 increased the expression of cytoplasmic p65 compared with the CLP + vehicle group, and Boc-2 reduced the effects of BML-111. **(C,D)** Nuclear p65 was up-regulated after CLP when compared with the sham group. BML-111 reversed the change in nuclear p65 level, and Boc-2 reduced the effects of BML-111. **(E,F)** Ac- NF-κB was increased in CLP group compared with sham group, BML-111 decreased the level of Ac- NF-κB than in CLP + Vehicle group, and Boc-2 reduced the effect of BML-111. **(G,H)** Sepsis significantly reduced the expression levels of SIRT1 in the cortex and hippocampus when compared with the sham group. BML-111 recovered the expression of SIRT1 compared with the CLP + vehicle group, and Boc-2 reduced the effects of BML-111. Data were shown as the means ± SEM (*n* = 3 surviving mice in **A**, *n* = 4 surviving mice in **B**, *n* = 5 surviving mice in **C**, *n* = 6 surviving mice in **D**, *n* = 3 surviving mice in **E,F**, *n* = 5 surviving mice in **G**, *n* = 6 surviving mice in **H**). ^∗^*P* < 0.05, ^∗∗^*P* < 0.01, ^∗∗∗^*P* < 0.001 represent CLP + Vehicle vs. Sham, ^#^*P* < 0.01, ^##^*P* < 0.01, ^###^*P* < 0.001 represent CLP + BML-111 vs. CLP + Vehicle, ^$^*P* < 0.05, ^$$^*P* < 0.01, ^$$$^*P* < 0.01 represent CLP + BML-111 + Boc-2 vs. CLP+BML-111.

To further explore the mechanism involved in sepsis-induced inflammation in the brain, we quantified the protein expression levels of cytoplasmic p65 and nuclear p65 in samples extracted from the cortex and hippocampus. The *in vivo* investigation revealed that the sepsis model led to a significantly decrease in cytoplasmic p65 relative to the sham group (*P* < 0.001, **Figure 8A**; and *P* < 0.01, **Figure 8B**). Meanwhile, neuroinflammation caused by sepsis significantly up-regulated the protein levels of nuclear p65 relative to the control group (*P* < 0.05, **Figure 8C**; and *P* < 0.01, **Figure 8D**). A particularly notable observation was that BML-111 treatment significantly increased the expression of cytoplasmic p65 relative to the CLP + vehicle group (*P* < 0.01, **Figure 8A**; and *P* < 0.05, **Figure 8B**) and decreased the expression of nuclear p65 relative to the CLP + vehicle group (*P* < 0.05, **Figure 8C**; and *P* < 0.001, **Figure 8D**). When Boc-2 was administered before CLP and BML-111 treatment, the protein levels of cytoplasmic p65 was significantly reduced relative to the CLP + BML-111 group (*P* < 0.05, **Figure 8A**; and *P* < 0.05, **Figure 8B**) and the protein level of nuclear p65 was significantly increased relative to the BML-111-treatment group (*P* < 0.001, **Figure 8C**; and *P* < 0.05, **Figure 8D**). Additional, neuroinflammation also increased the protein levels of Ac- NF-κB compared with sham group (*P* < 0.05, **Figure 8E**; and *P* < 0.05, **Figure 8F**). BML-111 treatment significantly decreased the expression of Ac- NF-κB relative to the CLP + vehicle group (*P* < 0.01, **Figure 8E**; and *P* < 0.01, **Figure 8F**). Administrating Boc-2 before CLP and BML-111 treatment was effective at increasing the protein levels of Ac- NF-κB relative to the BML-11- treatment group (*P* < 0.01, **Figure 8E**; and *P* < 0.01, **Figure 8F**).

### Sepsis-Induced the Down-Regulated SIRT1 Was Reduced by BML-111 Post-Treatment

It has been reported previously that, SIRT1 exerts protective effects in sepsis (Gao et al., 2015). We therefore used Western blotting to detect changes in SIRT1 in the cortex and hippocampus. **Figure 8G** and 8H showed that CLP decreased the SIRT1 level in the cortex and hippocampus relative to the sham group (*P* < 0.001, **Figure 8G**; and *P* < 0.001, **Figure 8H**). As expected, post-treatment with BML-111 up-regulated the expression of SIRT1 relative to the CLP + vehicle group (*P* < 0.05, **Figure 8G**; and *P* < 0.05, **Figure 8H**). Administering Boc-2 before CLP and BML-111 treatment down-regulated the expression of SIRT1 relative to the BML-111-treatment group (*P* < 0.05, **Figure 8G**; and *P* < 0.001, **Figure 8H**).

### Sepsis-Induced Neuroinflammation and Cognitive Impairment Were Ameliorated by BML-111 via the SIRT1/NF-κB Signaling Pathway

**FIGURE 9:**
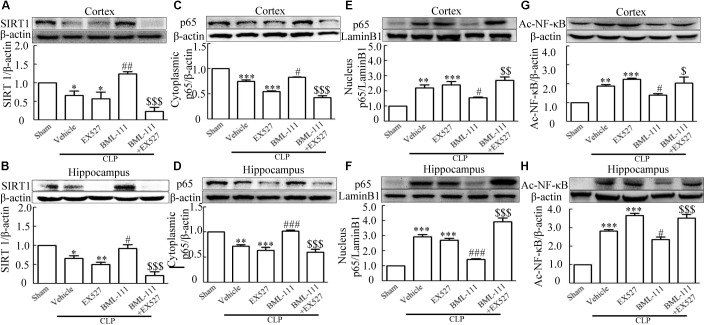
BML-111 inhibits NF-κB signaling pathway via SIRT1 in CLP mice. CLP and EX527 reduced the levels of SIRT1 **(A,B)** and cytoplasmic p65 **(C,D)** and increased the levels of nuclear p65 **(E,F)** and Ac-NF-κB **(G,H)** in the cortex and hippocampus when compared with the sham group. There was no significantly difference between CLP + Vehicle group and CLP + EX527 group. BML-111 increased the levels of SIRT1 **(A,B)** and cytoplasmic p65 **(C,D)** and decreased the levels of nuclear p65 **(E,F)** and Ac-NF-κB **(G,H)** compared with CLP + Vehicle group. EX527 administration before CLP and BML-111 reduced the levels of SIRT1 **(A,B)** and cytoplasmic p65 **(C,D)** and increased the levels of nuclear p65 **(E,F)** and Ac-NF-κB **(G,H)** when compared with the CLP + BML-111 group. Data were shown as the means ± SEM (*n* = 3 surviving mice in **A**, *n* = 5 surviving mice in **B**, *n* = 6 surviving mice in **C**, *n* = 5 surviving mice in **D**, *n* = 4 surviving mice in **E**, *n* = 6 surviving mice in **F**, *n* = 3 surviving mice in **G,H**). ^∗^*P* < 0.05, ^∗∗^*P* < 0.01, ^∗∗∗^*P* < 0.001 represent CLP + Vehicle vs. Sham and CLP + EX527 vs. sham, ^#^*P* < 0.05, ^##^*P* < 0.01, ^###^*P* < 0.001 represent CLP + BML-111 vs. CLP + Vehicle, ^$^*P* < 0.05, ^$$^*P* < 0.01, ^$$$^*P* < 0.01 represent CLP + BML-111 + EX527 vs. CLP + BML-111.

**FIGURE 10:**
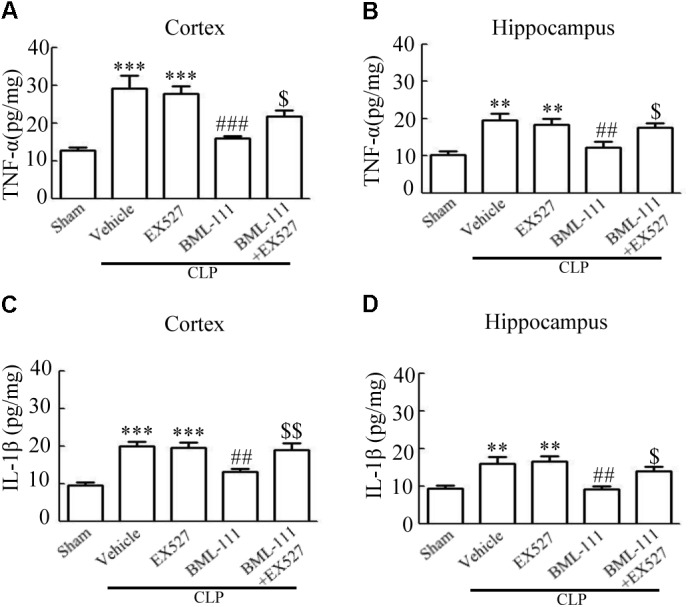
BML-111 inhibits the expression of inflammatory cytokines via SIRT1 in CLP mice. The expression levels of TNF-α and IL-1β were increased significantly in CLP + Vehicle and CLP + EX527 group when compared with sham group in cortex and hippocampus, and there was no difference in CLP + Vehicle group and CLP + EX527 group. BML-111 significantly decreased the levels of TNF-α and IL-1β compared with CLP + Vehicle group. EX527 up-regulated the expression of inflammatory cytokines in the cortex and hippocampus when compared with the CLP + BML-111 group. Data were shown as the means ± SEM (*n* = 5 surviving mice in **A–C**, *n* = 4 surviving mice in **D**). ^∗∗^*P* < 0.01, ^∗∗∗^*P* < 0.001 represent CLP + Vehicle vs. Sham and CLP + EX527 vs. sham, ^##^*P* < 0.01, ^###^*P* < 0.001 represent CLP + BML-111 vs. CLP + Vehicle, ^$^*P* < 0.05, ^$$^*P* < 0.01 represent CLP + BML-111 + EX527 vs. CLP + BML-111.

**FIGURE 11:**
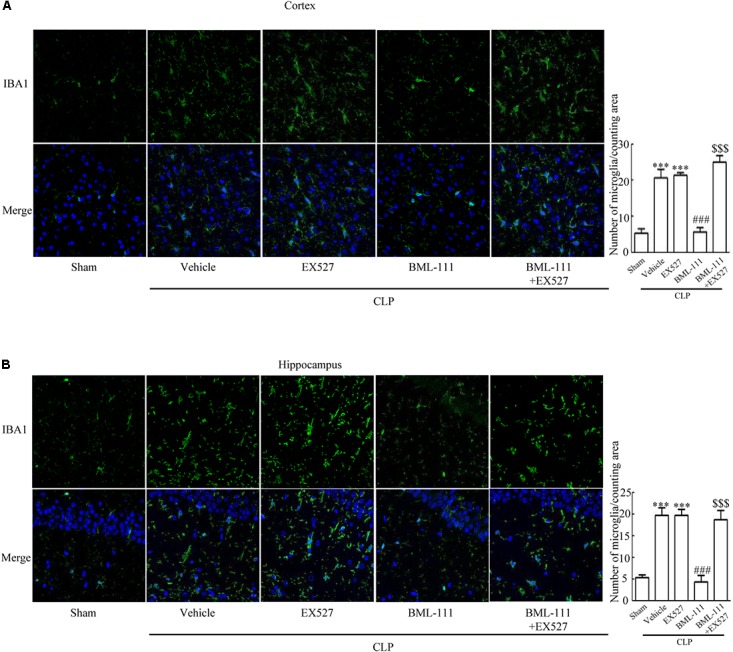
BML-111 decreases the activation of microglia induced via SIRT1 in CLP. **(A,B)** CLP and EX527 increased the activated microglia compared with sham group, and there did not differ between CLP + Vehicle group and CLP + EX527 group. BML-111 decreased the activated-microglia than in CLP + Vehicle group. EX527 treatment before CLP and BML-111 increased the activated microglia when compared with the CLP + BML-111 group. Scale bars indicate 50 μm (*n* = 3 surviving mice per group). ^∗∗∗^*P* < 0.001 represent CLP + Vehicle vs. Sham and CLP + EX527 vs. sham, ^###^*P* < 0.001 represent CLP + BML-111 vs. CLP + Vehicle,^$$$^*P* < 0.001 represent CLP + BML-111 + EX527 vs. CLP + BML-111.

**FIGURE 12:**
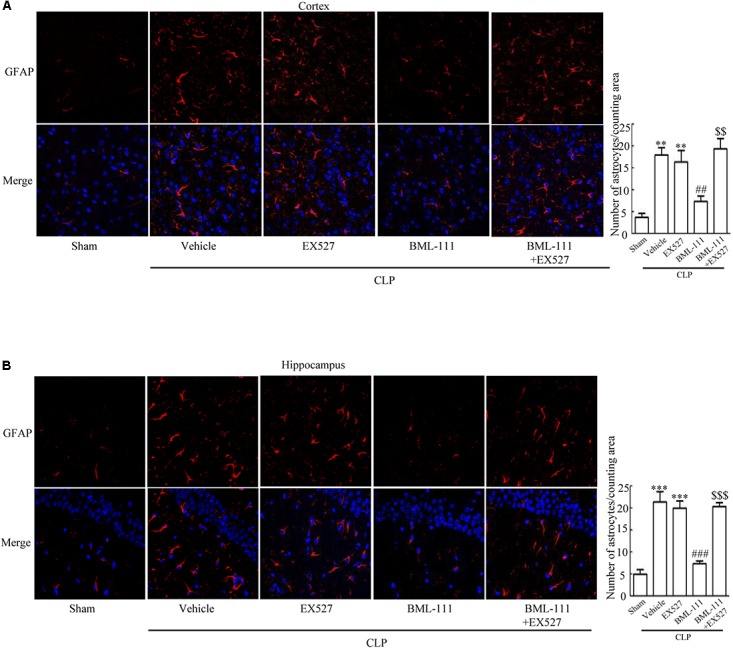
BML-111 decreases the activation of astrocyte induced via SIRT1 in CLP. **(A,B)** CLP and EX527 increased the activated astrocyte when compared with sham group, and there did not differ between CLP + Vehicle group and CLP + EX527 group. BML-111 decreased the activated astrocyte related to CLP + Vehicle group. EX527 administration before CLP and BML-111 increased the activation of astrocyte compared with the CLP + BML-111 group. Scale bars indicate 50 μm (*n* = 3 surviving mice per group). ^∗∗^*P* < 0.01, ^∗∗∗^*P* < 0.001 represent CLP + Vehicle vs. Sham and CLP + EX527 vs. sham, ^##^*P* < 0.01, ^###^*P* < 0.001 represent CLP + BML-111 vs. CLP + Vehicle,^$$^*P* < 0.01, ^$$$^*P* < 0.001 represent CLP + BML-111 + EX527 vs. CLP+BML-111.

**FIGURE 13:**
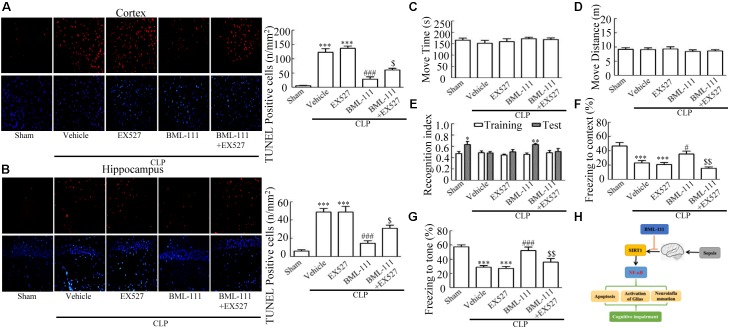
BML-111 decreases the number of TUNEL-positive cells and cognitive impairment in CLP. **(A,B)** The number of TUNEL-positive cells in cortex and hippocampus were significantly increased in CLP + Vehicle and CLP + EX527 mice compared with the sham group. BML-111 treatment reversed this variation in CLP + Vehicle and CLP + EX527 mice, and EX527 administration prior to CLP and BML-111 reduced these effects. Scale bars indicate 20 mm (*n* = 3 surviving mice per group). **(E–G)** EX527 treatment before CLP and BML-111 treatment decreased the recognition index for the new object when compared with the familiar one and decreased the freezing behaviors compared with the CLP + BML-111 group. **(C,D)** Move time and distance in the open-field-test. There was no difference among groups. **(H)** Schematic overview showing the involvement of BML-111 in sepsis-induced brain dysfunction. Data were shown as the means ± SEM (*n* = 10 survived mice in **C–G**). ^∗^*P* < 0.05, ^∗∗^*P* < 0.01 represent Test vs. Training in **E**. ^∗∗∗^*P* < 0.001 represent CLP + Vehicle vs. Sham and CLP + EX527 vs. sham in **A,B,F,G**, ^#^*P* < 0.05, ^###^*P* < 0.001 represent CLP + BML-111 vs. CLP + Vehicle in **A,B,F,G**, ^$^*P* < 0.05, ^$$^*P* < 0.01 represent CLP + BML-111 + EX527 vs. CLP + BML-111 in **A,B,F,G**.

These results together with those reported in the literature prompted us to explore the relationships between SIRT1 and NF-κB and the mechanism underlying the effects of BML-111. EX527, which is an antagonist of SIRT1, was utilized in the experiments. As **Figures 9A,B** shows, the protein levels of SIRT1 decreased in CLP + Vehicle group and CLP + EX527 group relative to the sham group (*P* < 0.05, *P* < 0.05, **Figure 9A**; and *P* < 0.05, *P* < 0.05, **Figure 9B**). And there was no differ in CLP + Vehicle group and CLP + EX527 group (*P* > 0.05, **Figures 9A,B**) Administering BML-111 increased the protein levels of SIRT1 relative to the CLP + Vehicle group (*P* < 0.01, **Figure 9A**; and *P* < 0.05, **Figure 9B**). EX527 significantly inhibited the expression level of SIRT1 relative to the CLP + BML-111 group (*P* < 0.001, **Figure 9A**; and *P* < 0.001, **Figure 9B**). A previous study (Lei et al., 2012) found that the activation of SIRT1 reduced the migration of cytoplasmic p65 to the nucleus, and so we also explored variations induced by the administration of EX527. As shown in **Figures 9C–F**, CLP + Vehicle and CLP + EX527 down-regulated cytoplasmic p65 and up-regulated nuclear p65 in the cortex and hippocampus relative to the control group (*P* < 0.001, *P* < 0.001, **Figure 9C**; *P* < 0.01, *P* < 0.001, **Figure 9D**; *P* < 0.01, *P* < 0.001, **Figure 9E**; and *P* < 0.001, *P* < 0.001, **Figure 9F**). In addition, the protein levels of cytoplasmic p65 and nuclear p65 did not differ in CLP + Vehicle group and CLP + EX527 group (*P* > 0.05, **Figures 9C–F**). BML-111 up-regulated cytoplasmic p65 and down-regulated nuclear p65 relative to CLP + Vehicle group (*P* < 0.05, **Figure 9C**; *P* < 0.001, **Figure 9D**; *P* < 0.05, **Figure 9E**; and *P* < 0.001, **Figure 9F**). EX527 down-regulated cytoplasmic p65 and up-regulated nuclear p65 relative to CLP + BML-111 group (*P* < 0.001, **Figure 9C**; *P* < 0.001, **Figure 9D**; *P* < 0.01, **Figure 9E**; and *P* < 0.001, **Figure 9F**). At the same time, the protein levels of Ac- NF-κB in CLP + Vehicle and CLP + EX527 group increased significantly compared with sham group (*P* < 0.01, *P* < 0.001, **Figure 9G**; *P* < 0.001, *P* < 0.001, **Figure 9H**). The protein levels of Ac- NF-κB was no difference in CLP + Vehicle and CLP + EX527 group (*P* > 0.05, **Figures 9G,H**). BML-111 treatment down-regulated Ac- NF-κB relative to CLP + Vehicle group (*P* < 0.05, **Figure 9G**; *P* < 0.05, **Figure 9H**). EX527 increased Ac- NF-κB relative to CLP + BML-111 group (*P* < 0.05, **Figure 9G**; *P* < 0.001, **Figure 9H**). The expression levels of TNF-α and IL-1β in the cortex and hippocampus were increased in CLP + Vehicle group and CLP + EX527 group relative to sham group (*P* < 0.001, *P* < 0.001, **Figure 10A**; *P* < 0.01, *P* < 0.01, **Figure 10B**; *P* < 0.001, *P* < 0.001, **Figure 10C**; and *P* < 0.01, *P* < 0.01, **Figure 10D**). And TNF-α and IL-1β in the cortex and hippocampus did not differ in CLP + Vehicle group and CLP + EX527 group (*P* > 0.05, **Figures 10A–D**). Administering BML-111 significantly reduced the levels of TNF-α and IL-1β relative to mice subjected to CLP (*P* < 0.001, **Figure 10A**; *P* < 0.01, **Figure 10B**; *P* < 0.01, **Figure 10C**; and *P* < 0.01, **Figure 10D**). EX527 increased the levels of TNF-α and IL-1β in the cortex and hippocampus relative to the CLP + BML-111 group (*P* < 0.05, **Figure 10A**; *P* < 0.05, **Figure 10B**; *P* < 0.01, **Figure 10C**; and *P* < 0.05, **Figure 10D**). CLP and EX527 significantly increased the numbers of activated microglia and astrocyte in cortex and hippocampus compared with sham group (*P* < 0.001, *P* < 0.001, **Figure 11A**; *P* < 0.001, *P* < 0.001, **Figure 11B**; *P* < 0.01, *P* < 0.01, **Figure 12A**; *P* < 0.001, *P* < 0.001, **Figure 12B**). Administering BML-111 decreased the cells than in CLP + Vehicle group (*P* < 0.001, **Figure 11A**; *P* < 0.001, **Figure 11B**; *P* < 0.01, **Figure 12A**; *P* < 0.001, **Figure 12B**). And pretreatment with EX527 increased the numbers of cells relative to the CLP + BML-111 group (*P* < 0.001, **Figure 11A**; *P* < 0.001, **Figure 11B**; *P* < 0.01, **Figure 12A**; *P* < 0.001, **Figure 12B**). There were significantly more TUNEL-positive cells in the cortex and hippocampus in the CLP + Vehicle group and CLP + EX527 group than in the sham group (*P* < 0.001, *P* < 0.001, **Figure 13A**; *P* < 0.001, *P* < 0.001, **Figure 13B**). BML-111 decreased the number of TUNEL-positive cells compared with CLP + Vehicle group (*P* < 0.001, **Figure 13A**; *P* < 0.001, **Figure 13B**), while pretreatment with EX527 increased the number of TUNEL-positive cells compared with BML-111 treatment alone (*P* < 0.05, **Figure 13A**; *P* < 0.05, **Figure 13B**).

Finally, the survivors underwent behavioral open-field, novel-object-recognition, and fear-conditioning tests. The recognition index of sham group for the new object was differing significantly from that for the familiar one (*P* < 0.05, **Figure 13E**). CLP and EX527 made the recognition index of the new object did not differ from that for the familiar one (*P* > 0.05, **Figure 13E**). Animals in the BML-111-treatment group spent more time investigating the new object, leading to the recognition index for the new object differing significantly from that for the familiar one (*P* < 0.01, **Figure 13E**). Mice in the CLP + BML-111 + EX527 group spent less time investigating the new object, resulting in the recognition index for the new object not differing from that for the familiar one (*P* > 0.05, **Figure 13E**). During fear-conditioning test, CLP and EX527 decreased the times to context and tone significantly than in sham group (*P* < 0.001, *P* < 0.001, **Figure 13F**; *P* < 0.001, *P* < 0.001, **Figure 13G**). And there were no difference in CLP + Vehicle group and CLP + EX527 group (*P* > 0.05, **Figures 13F,G**). BML-111 increased the freezing time compared with CLP + Vehicle group (*P* < 0.05, **Figure 13F**; *P* < 0.001, **Figure 13G**). Administering EX527 prior to CLP and BML-111 treatment resulted in the freezing time decreasing compared with that in the CLP + BML-111 group (*P* < 0.01 for both, **Figures 13F,G**). As expected, there were no significant difference in the move time or distance (*P* > 0.05 for both, **Figures 13C,D**). The working hypothesis of the study had been presented in **Figure 13H**. Cognitive impairment is a major complication of Sepsis. The apoptosis of neurons caused by neuroinflammation is one important mechanism of cognitive impairment induced by sepsis. Neuroinflammation could be elicited due to activated NF-κB signaling pathway followed by decreased SIRT1. BML-111 could increase the level of SIRT1 and then inhibit the activation of NF-κB signaling pathway, which at last alleviated the activation of glias, mitigated neuroinflammation, decreased the number of apoptosis cells and improved cognitive impairment (**Figure 13H**).

## Discussion

The aim of this study was to determine whether BML-111 can improve neuroinflammation and cognitive impairment in sepsis via the SIRT1/NF-κB signaling pathway. The main findings of our study were that (a) BML-111 reduced the level of neuroinflammation, activation of glias, number of TUNEL-positive cells, and cognitive impairment induced by sepsis, and (b) the underlying mechanism was activation of SIRT1 by BML-111 followed by suppression of the NF-κB activation.

Cognitive impairment is a severe complication of sepsis that manifests clinically as delirium or even coma. Sepsis has been suggested to be related to the cognitive impairment (Adam et al., 2013), that often presents in patients recovering from sepsis and results in a lower quality of life (Gofton and Young, 2012; Sankowski et al., 2015). A previous study identified that severe sepsis induced by CLP could cause cognitive impairment, and our results confirm the previous finding that mice that survive severe sepsis can exhibit cognitive impairment (Wu et al., 2015). Meanwhile, our open-field test data suggested that the locomotor and exploratory abilities were not affected by the surgical procedure, which was consistent with previous work (Wei et al., 2015). Alterations in learning and memory are the two main manifestations of cognitive impairment. Our results suggest that CLP induced cognitive impairment in both the novel-object-recognition and fear-conditioning tests, which is also consistent with previous findings (Ji et al., 2015; Wei et al., 2015). A previous study (Tang et al., 2016) suggested that BML-111 at 1 mg/kg could reduce systemic inflammatory responses and protect the lungs. We explored whether BML-111 at lower doses (0.01 and 0.1 mg/kg) administered via intracerebroventricular injections could reduce neuroinflammation and protect the brain during sepsis, and found that BML-111 at a dose of 0.1 mg/kg could reduce the cognitive impairment induced by sepsis.

The mechanism underlying cognitive impairment has been explored widely, but a specific therapy for this syndrome remains to be identified (Adam et al., 2013). The apoptosis of neurons caused by neuroinflammation has been suggested to be an important mechanism underlying the cognitive impairment induced by sepsis (Liu et al., 2014; Ning et al., 2017). Previous studies found that neuroinflammation induced by sepsis increased the activation of glias and the apoptosis of neurons, leading to cognitive impairment (Semmler et al., 2005, 2007; Lemstra et al., 2007; Adam et al., 2013; Wei et al., 2015; Zhao et al., 2015). The present data suggest that the levels of inflammatory mediators such as TNF-α and IL-1β increase sharply in the cortex and hippocampus after CLP, demonstrating that CLP can induce neuroinflammation. Using markers of microglias and astrocytes (Iba1 and GFAP), we found that glias were activated in the cortex and hippocampus during sepsis. The number of TUNEL-positive cells was markedly increased in the cortex and hippocampus in CLP mice. Combining these observations with our behavioral findings, we suggest that mice with sepsis develop cognitive impairment due to neuronal apoptosis induced by neuroinflammation. Other studies have also shown that BML-111 can resolve inflammation in the lungs and brain (Gong J. et al., 2012; Hawkins et al., 2014). The findings of the present study suggest that BML-111 markedly decreases the levels of inflammatory cytokines without changing the level of TNF-α in the blood, and also reduces the activation of glias, the number of TUNEL-positive cells, and cognitive impairment in the brains during sepsis. We therefore suggest that BML-111 can reduce neuroinflammation and cognitive impairment without affecting the systemic inflammatory response.

As a synthetic ALX agonist, BML-111 could mitigate inflammatory response in the lung and intestinal mucosal barrier during sepsis by inhibiting the activation of the Akt, ERK1/2, and p38 MAPK signaling pathways and decreasing the expression of TLR2 and TLR4 (Liu H. et al., 2015; Tang et al., 2016). Neuroinflammation induced by middle cerebral artery occlusion (MCAO) could be restrained after administration BML-111 by decreasing the activation of microglia (Hawkins et al., 2014). However, these benefits were obtained by intraperitoneal and intravenous injections. BML-111 was injected intracerebroventricularly in order to illuminate the molecular mechanism of effect of BML-111 in neuroinflammation and cognitive impairment induced by sepsis. In our study, BML-111 up-regulated the level of SIRT1 and then suppressed the activation of NF-κB and deacetylation of NF-κB p65, which subsequently decreased the activation of microglia, reduced neuroinflammation, improved apoptosis cells and cognitive impairment. SIRT1 is reported to play a critical role in the sepsis. SIRT1 protects mitochondrial membrane potential and organ dysfunction by activation of the PGC1alpha and NFE2L2 pathways (McCreath et al., 2016). Importantly, SIRT1 down-regulated the level of Ac- NF-κB expression and then decreased the expression of pro-inflammatory cytokines (Zhao et al., 2015). In the current data, BML-111 alleviated neuroinflammation simultaneously did not affect the periphery inflammatory response. In other words, BML-111 could protect brain before sepsis was improved, which might improve the prognosis of cognitive impairment induced by sepsis. Therefore, SIRT1, as a key target in this important inflammatory signaling pathway, will also be a crucial target in the treatment of brain dysfunction induced by sepsis. Our present study suggested that BML-111 might be a pharmacological template for the treatment of sepsis.

Lipoxin A4 (LXA4), the endogenous molecule, could promote inflammatory resolution through inhibiting pro-inflammation signaling pathways including NF-κB. But, the native LXA4 is rapidly inactivated *in vivo*. Though it has been reported that the activation of SIRT1 induced by resveratrol could augment the production of lipoxin A4 in some diseases, such as autism, obesity, diabetes mellitus, metabolic syndrome, depression, schizophrenia, and cancer (Diaz-Gerevini et al., 2016), the interrelationship of LAX4 and SIRT1 in sepsis induced by brain dysfunction need to be illustrated. In this study, we did not design experiments to explore the relationship between LXA4 and SIRT1 in brain dysfunction induced by sepsis, but it does have the potential to become a novel point in our future studies.

While the present data are encouraging, this study was subject to a few limitations. Firstly, we used the open-field, novel-object-recognition, and fear-conditioning tests to reflect behavioral alterations associated with sepsis, and so other behavioral tests such as the Morris water maze task should be included in future studies. Secondly,the dose and administration route of BML-111 in this study differed from those used in other studies, and so further studies are needed to accurately determine the optimal dose and mode of administration.

## Conclusion

Administering BML-111 reduced cognitive impairment in survivors of sepsis. The protective mechanism might involve reductions in neuroinflammatory responses, the activation of glias, and the number of TUNEL-positive cells via effects on the SIRT1/NF-κB signaling pathway.

## Author Contributions

SP and YWu made equal contribution to this work, performed research, analyzed data, and wrote the manuscript. YS designed the research. SY revised and edited the manuscript. All authors read and approved the final manuscript.

## Conflict of Interest Statement

The authors declare that the research was conducted in the absence of any commercial or financial relationships that could be construed as a potential conflict of interest.

## Supplementary Material

FIGURE S1 BML-111 administered via intracerebroventricular injection does not affect the peripheral inflammatory response. Sepsis increased the level of TNF-α in the blood, and this was not affected by BML-111 injection. Data were shown as the means ± SEM (*n* = 6 survived mice per group).
