# Supplementary figures and images for: BML-111 Reduces Neuroinflammation and Cognitive Impairment in Mice With Sepsis via the SIRT1/NF-κB Signaling Pathway
Source: Front Cell Neurosci. 2018 Aug 21;12:267. doi: 10.3389/fncel.2018.00267 (PMC6110933; doi:10.3389/fncel.2018.00267)

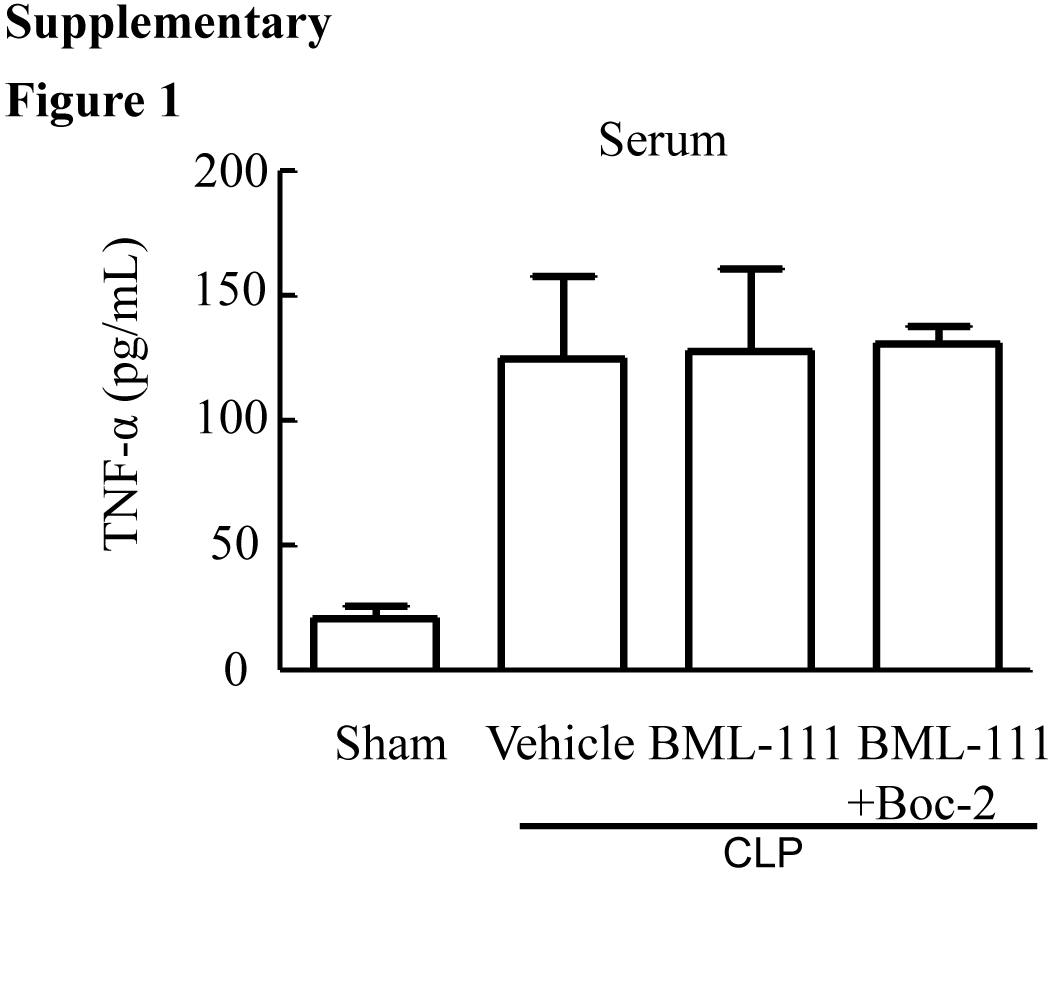

Supplement: FIGURE S1 — BML-111 administered via intracerebroventricular injection does not affect the peripheral inflammatory response. Sepsis increased the level of TNF-α in the blood, and this was not affected by BML-111 injection. Data were shown as the means ± SEM (n = 6 survived mice per group). [file Image_1.TIF]
